# Duration-dependent gut microbiota disruption drives opposing effects on adipose thermogenesis in obese mice

**DOI:** 10.3389/fmicb.2026.1860759

**Published:** 2026-07-22

**Authors:** Tianle Feng, Yuanjie Cui, Xinwen Bi, Yang Liu, Lian Yang, Dongyue Ouyang, Weixin Xu, Yanyan Zhao, Yuqin Xi, Qiong Yang, Yixin Sun, Xue Wang, Ming Li

**Affiliations:** West China School of Public Health and West China Fourth Hospital, Sichuan University, Chengdu, China

**Keywords:** adipose thermogenesis, antibiotic, browning, duration-dependent effects, energy expenditure, gut microbiota, obesity

## Abstract

**Background:**

Antibiotic-induced disruption of the gut microbiota is known to influence host metabolism; however, whether its metabolic effects depend on the duration of exposure remains unclear, particularly in relation to adipose thermogenesis under high-fat diet (HFD) conditions.

**Methods:**

C57BL/6 mice fed an HFD were treated with a broad-spectrum antibiotic cocktail for either short-term (2 weeks) or long-term (10 weeks). Body weight, adiposity, metabolic parameters, and energy expenditure were assessed. Thermogenic capacity was evaluated by histology and gene expression analysis in brown and white adipose tissues, following stimulation with the β3-adrenergic receptor agonist CL316243. Gut microbiota composition was analyzed using 16S rRNA sequencing.

**Results:**

Short-term antibiotic treatment attenuated HFD-induced weight gain, reduced fat accumulation, and increased weight-adjusted CL316243-stimulated metabolic rate, accompanied by upregulation of thermogenic genes (*Ppargc1a*, *Pparg*, *Ucp1*). In contrast, long-term treatment exacerbated obesity, decreased metabolic rate and rectal temperature, and suppressed thermogenic gene expression. Microbiota analysis revealed partial recovery of *α*-diversity after short-term intervention, whereas long-term treatment led to persistent dysbiosis with reduced diversity and altered community structure.

**Conclusion:**

The metabolic consequences of antibiotic-induced microbiota disruption are strongly duration-dependent. Transient perturbation is associated with enhanced adipose thermogenesis and energy expenditure, whereas prolonged disruption correlates with impaired thermogenic capacity and worsened metabolic outcomes. However, causal relationships remain to be established. These findings highlight the importance of temporal dynamics in host–microbiota interactions and provide insight into microbiota-targeted strategies for metabolic disorders.

## 1 Introduction

Obesity, characterized by excessive fat accumulation resulting from a chronic imbalance between energy intake and expenditure (Hill et al., 2012), has become a major global public health challenge (NCD Risk Factor Collaboration (NCD-RisC), 2024). It is strongly associated with an elevated risk of multiple metabolic disorders, including type 2 diabetes, cardiovascular diseases, and non-alcoholic fatty liver disease (Powell-Wiley et al., 2021; Banerjee and Mani, 2025). While reducing caloric intake and increasing physical activity remain the primary strategies for weight management, approaches aimed at enhancing the intrinsic energy expenditure mechanisms may offer a promising therapeutic avenue (Betz and Enerbäck, 2018).

In mammals, adaptive thermogenesis serves as a key pathway for energy dissipation and is primarily mediated by adipose tissue. Brown adipose tissue (BAT) is a specialized thermogenic tissue that generates heat through the action of mitochondrial uncoupling protein 1 (UCP1) (Chouchani et al., 2019). Additionally, specific depots of white adipose tissue (WAT) can undergo a phenotypic conversion which is termed “browning” or “beiging,” wherein the adipocytes acquire characteristics of BAT. This transition is marked by increased expression of UCP1 and enhanced mitochondrial biogenesis (Zheng et al., 2023). Consequently, beige adipose tissue contributes substantially to whole-body energy expenditure (Harms and Seale, 2013; Rosen and Spiegelman, 2014). The transcriptional regulation of this process involves key factors such as peroxisome proliferator-activated receptor gamma (PPAR-gamma), its coactivator PGC-1alpha, and UCP1 (Takeda et al., 2023; Yang et al., 2024), while receptor-interacting protein 140 (RIP140) acts as a transcriptional corepressor (Tsagkaraki et al., 2023). Given the thermogenic potential shared by both classical BAT and induced beige adipocytes, promoting BAT activity and WAT browning may represent a potential strategy to counteract obesity by enhancing energy expenditure.

The gut microbiota has emerged as a critical environmental determinant of host energy homeostasis and metabolic health, thereby establishing its role as an additional contributing factor in the pathophysiology of obesity (Turnbaugh et al., 2006). Dysbiosis, a disruption of this delicate microbial equilibrium, has been firmly implicated in obesity and metabolic syndrome across both human and animal models (Bäckhed et al., 2004; Tremaroli and Bäckhed, 2012). A common source of such disruption is antibiotic intervention, which drastically alters gut microbiota composition, and exerts complex, context-dependent effects on host metabolism. Current evidence remains conflicted regarding the metabolic consequences of antibiotic-induced dysbiosis. Some studies indicate that such dysbiosis exacerbates diet-induced obesity and impairs glucose homeostasis (Cox et al., 2014), manifesting as aggravated insulin resistance and glucose intolerance (Mahana et al., 2016; Rodrigues et al., 2017). Mechanistically, antibiotic-induced depletion suppresses the induction of *Ucp1* expression and impairs WAT browning, thereby diminishing the thermogenic capacity of BAT (Li et al., 2019). Yet others report that antibiotic treatment may actually alleviate high-fat diet (HFD)-induced obesity by modulating glucose and lipid metabolism (Fujisaka et al., 2016; Luo et al., 2023), improving insulin sensitivity, and promoting the development of functional beige adipose tissue (Suárez-Zamorano et al., 2015). However, these studies largely differ in the duration of antibiotic exposure, a variable that has not been systematically examined. Most employ a single, discrete window of antibiotic exposure: short-term (2–4 weeks), long-term (8–10 weeks), or continuous treatment from gestation onward. Such differences in exposure protocol directly contribute to divergent or even opposite metabolic outcomes, including effects on adiposity, insulin resistance, and BAT thermogenesis.

To clarify this relationship, we employed a broad-spectrum antibiotic cocktail to perturb the gut microbiota in HFD-fed C57BL/6 mice over different durations. We aimed to investigate: (1) the effects of short-term versus long-term antibiotic-induced dysbiosis on the development of HFD-induced obesity; (2) the consequent changes in systemic energy metabolism indicators; and (3) the alterations in BAT activity and WAT browning potential at the gene expression level. We hypothesized that the gut microbiota may influence obesity progression by modulating the thermogenic function of adipose tissues, thereby affecting systemic energy expenditure. This study provides new insights into the “gut microbiota-adipose tissue axis” and its role in energy metabolic regulation during obesity.

## 2 Materials and methods

### 2.1 Animals and diet

A total of 100 C57BL/6 male mice (10-week-old) were purchased from SPF Biotechnology Co., Ltd. (Beijing, China). The mice were housed under controlled conditions (12/12 h light/day cycle, 25 ± 2 °C, relative humidity of 60 ± 10%) with free access to food and water. The high-fat diet provided 60.0% of total caloric intake from fat, with carbohydrates and protein providing 25.9 and 14.1% of energy, respectively, totaling 5.50 kcal/g. The standard chow derived 11% of its calories from fat, with carbohydrates and protein providing 66.0 and 23.0% of energy, totaling 2.40 kcal/g. At the end of the experiment, all mice were euthanized by cervical dislocation performed by trained personnel. Mice were randomly assigned to experimental groups using a computer-generated random number sequence (Excel Rand function). No animals were excluded from the study; all 100 mice completed the 10-week experiment without signs of severe illness or mortality. This experiment was approved by the Ethics Committee of West China Fourth Hospital/West China School of Public Health, Sichuan University (Approval No. Gwll2023232).

### 2.2 Antibiotic treatment

A broad-spectrum antibiotic (ABX) cocktail was prepared containing ampicillin (100 mg/kg), neomycin (100 mg/kg), amphotericin B (1 mg/kg), bacitracin (100 mg/kg), vancomycin (50 mg/kg), and imipenem/cilastatin sodium (50 mg/kg) (Li et al., 2022). These doses represent the final administered amount per kilogram of body weight. To achieve accurate dosing by oral gavage, a stock solution was prepared as follows: based on an average mouse body weight of 25 g at the start of treatment, each antibiotic was dissolved in sterile saline to a concentration that would deliver the desired mg/kg dose in a reference volume of 0.2 mL per 25 g mouse. For example, for ampicillin (100 mg/kg), the concentration was calculated as (100 mg/kg × 0.025 kg)/(0.2 mL) = (12.5 mg/mL). During the daily gavage, the actual volume administered to each mouse was adjusted proportionally to its individual body weight on that day to maintain the exact mg/kg dose. Specifically, the gavage volume was calculated as:

Volume(mL)=(body weight in grams)×(0.2mL/25g).

For example, a 30 g mouse received 0.24 (mL). This protocol ensures that every animal receives the stated mg/kg dose regardless of weight variation.

### 2.3 Grouping and treatment

Mice were randomly assigned to five groups (*n* = 20 per group): Blank Group (BG; standard chow + saline), High-Fat Diet Group (FG; HFD + saline), Antibiotic Control Group (AG; standard chow + 2-week ABX), Short-term Antibiotic and HFD Group (S-AFG; HFD + 2-week ABX), and Long-term Antibiotic and HFD Group (L-AFG; HFD + 10-week ABX). After 1 week of acclimatization, the FG, S-AFG, and L-AFG groups were switched to HFD for 10 weeks, while BG and AG groups remained on standard chow. For the first 2 weeks, all antibiotic-treated groups (AG, S-AFG, L-AFG) received daily oral gavage of the antibiotic cocktail; the control groups (BG, FG) received daily oral gavage of sterile saline at the same volume (adjusted for body weight as described in section 2.2). From week 3 to week 10, only the L-AFG group continued antibiotic gavage every other day. All other groups (BG, FG, AG, S-AFG) received sterile saline gavage every other day at the same volume to match the gavage frequency and handling stress experienced by the L-AFG group. This sham-gavage procedure was performed using the same gavage needle and handling protocol as for antibiotic administration. At experimental week 10, all groups received intraperitoneal injections of the β3-adrenergic receptor agonist CL316243 (1 mg/kg) for 5 consecutive days to stimulate maximal thermogenic capacity.

### 2.4 Physiological and biochemical measurements

Body weight was measured weekly, and food intake was recorded over two consecutive days each week to estimate average daily intake. At the end of the experiment (week 10), mice were fasted for 8 h and fasting blood glucose was measured. These procedures were performed before euthanasia. After euthanasia, adipose tissues and major organs were collected and weighed; body fat percentage (BFP) and organ coefficients were calculated using standard formulas. Blood was collected from the retro-orbital vessels and centrifuged at 4 °C and 5,000 rpm for 10 min to obtain serum. The serum was then analyzed for triglycerides (TG), total cholesterol (TC), high-density lipoprotein cholesterol (HDL-C), and low-density lipoprotein cholesterol (LDL-C), using an automatic biochemical analyzer.

### 2.5 Thermogenic capacity assessments

For metabolic rate and rectal temperature measurements, eight mice per group (randomly selected from the 20) were used. To assess drug-stimulated thermogenic capacity, a flow-through indirect calorimetry system (TSE Systems, Germany) was used (Speakman, 2013). The system consists of a four-chamber open-circuit respirometry setup with integrated gas analyzers for oxygen consumption (VO₂) and carbon dioxide production (VCO₂) measurement. Each metabolic chamber (volume 4.9 L) was maintained at thermoneutrality for mice (30 ± 1 °C) using a temperature-controlled cabinet to minimize cold-induced thermogenesis. A 12 h light/dark cycle was maintained throughout the measurements. More details can be found in Supplementary Table 1. Rectal temperature was measured using a calibrated digital thermometer with a lubricated probe inserted to a standardized depth of 2 cm into the rectum. Measurements were performed in a quiet environment at a fixed time point (30–60 min after the final CL316243 injection) by the same operator to minimize handling stress and circadian variation. For histological analysis of WAT and BAT, all 20 mice per group were used. WAT and BAT samples were fixed in 4% (w/v) paraformaldehyde for 24 h, embedded in paraffin, and subjected to hematoxylin–eosin (HE) staining. WAT sections were cut at 5 μm and BAT sections at 3 μm. Adipocyte morphology was examined under a microscope, and cell diameters were measured using ImageJ software. For histological analysis, the investigator performing adipocyte diameter measurement (using ImageJ) was blinded to group allocation. Slides were coded by a technician not involved in the study, and the code was broken only after all measurements were completed.

### 2.6 RNA extraction and RT-qPCR

Total RNA was extracted from the remaining WAT and BAT samples using an Animal Total RNA Isolation Kit (FOREGENE) according to the manufacturer’s protocol. To prevent RNA degradation, tissues had been immersed in animal tissue storage solution and stored at −80 °C prior to extraction. For each sample, 500 ng of total RNA was reverse-transcribed into cDNA using a gDNA removal and first-strand cDNA synthesis kit. Quantitative real-time PCR (qPCR) was subsequently conducted with SYBR Green I Master Mix on a real-time PCR detection system. The mRNA levels of browning-related genes—uncoupling protein 1 (UCP1), peroxisome proliferator-activated receptor *γ* (PPAR-gamma), PPAR-gamma coactivator-1α (PGC-1alpha), and nuclear receptor interacting protein 1 (RIP140)—were quantified and normalized to GAPDH as an internal reference. Relative expression was calculated using the 2^−ΔΔC^ᵗ method. To assess mitochondrial abundance in WAT, mitochondrial DNA (mtDNA) copy number was determined by qPCR following DNA extraction with a Microscale Genomic DNA Extraction Kit, using *β*-actin as the reference gene (Shen et al., 2025). The mitochondrial target gene was MT-ND1 (mitochondrially encoded NADH dehydrogenase 1). All primer sequences are provided in Supplementary Table 2.

### 2.7 Gut microbiota sequencing

For microbiome analysis, ten mice per group (randomly selected from the 20) were used. Fecal samples were collected from these ten mice at two time points: at the end of the 2-week gavage period and at the termination of the experiment (week 10). Fresh feces were immediately sent to Chengdu Baseter Biotechnology Co., Ltd. for 16S rRNA amplicon sequencing on the Illumina NovaSeq 6,000 platform. Subsequent bioinformatics analyses included amplicon sequence variant (ASV) inference using the DADA2 algorithm, taxonomic annotation against the SILVA 138 database, and microbial diversity assessment at both the phylum and genus levels. All detailed parameters of 16S rRNA gene sequencing and bioinformatics analysis are summarized in Supplementary Table 3. To statistically assess differences in microbial community composition among groups, PERMANOVA (Permutational Multivariate Analysis of Variance) was performed using the “adonis2” function from the R package “vegan” based on Bray-Curtis distance matrices, with 999 permutations. Pairwise PERMANOVA comparisons were adjusted for multiple testing using the Benjamini-Hochberg false discovery rate (FDR) method. Additionally, ANOSIM (Analysis of Similarities) was performed using the “anosim” function in “vegan” with 999 permutations. All analyses were conducted in R (version 4.2.1).

### 2.8 Statistics and analysis

Data are presented as mean ± SD. Statistical analyses were performed using GraphPad Prism (version 10.2) and SPSS (version 23, IBM Corp., USA). Normality was assessed using the Shapiro–Wilk test. Comparisons between two groups were conducted using unpaired Student’s t-tests, while multiple group comparisons were performed using one-way ANOVA followed by Tukey’s *post hoc* test. For energy expenditure analysis, group differences were evaluated using analysis of covariance (ANCOVA) with body weight as a covariate. Non-parametric tests were applied when appropriate. A *p* value less than 0.05 was considered statistically significant.

## 3 Results

### 3.1 Transient antibiotic treatment mitigates, whereas its prolonged exposure exacerbates, HFD-induced obesity and metabolic dysfunction

**Figure 1:**
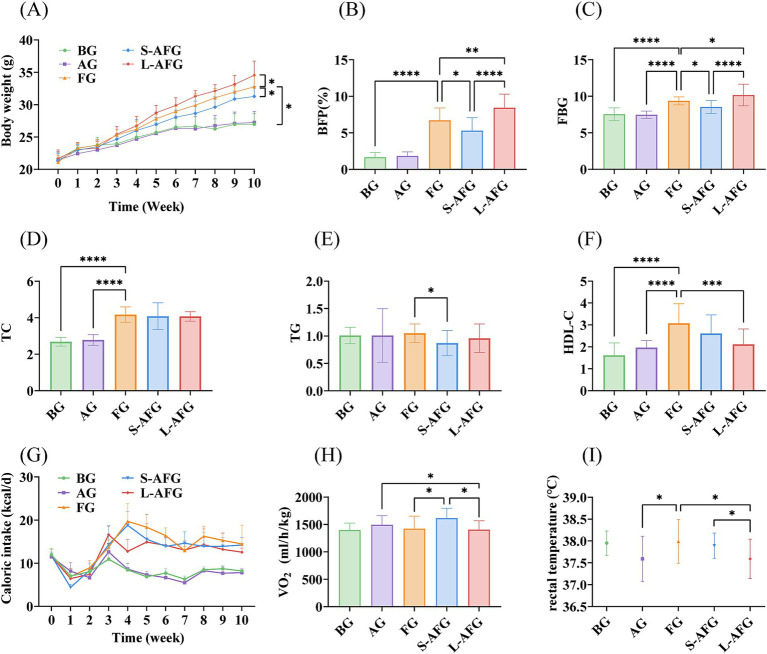
Short-term antibiotic perturbation mitigates, whereas long-term depletion exacerbates, HFD-induced obesity and metabolic dysfunction. **(A)** Body weight trajectories of mice over the 10-week experimental period. **(B)** Body fat percentage (BFP) at sacrifice. **(C–F)** Levels of fasting blood glucose (FBG), total cholesterol (TC), triglycerides (TG), and high-density lipoprotein cholesterol (HDL-C). **(G)** Average daily caloric intake per mouse. **(H)** Weight-adjusted CL316243-stimulated metabolic rate. **(I)** Rectal temperature measured 30–60 min after the final CL316243 injection. BG, blank group (standard chow + saline); FG, high-fat diet group (HFD + saline); AG, antibiotic control group (standard chow + 2-week antibiotics); S-AFG, short-term antibiotic and HFD group (HFD + 2-week antibiotics); L-AFG, long-term antibiotic and HFD group (HFD + 10-week antibiotics). For panels **(A–G)**, *n* = 20 mice per group. For panel **(H)** (metabolic rate) and panel **(I)** (rectal temperature), *n* = 8 mice per group. Data are expressed as the mean ± SD. *p < 0.05, **p < 0.01, and ***p < 0.001, ****p < 0.0001, versus the indicated groups.

After 4 weeks of feeding, mice in all three HFD groups showed a significant increase in body weight (approximately 20% higher than the control diet group, BG) (Figure 1A). Although all HFD-fed groups developed obesity, the degree of adiposity differed depending on antibiotic intervention. Body weight in the S-AFG group was reduced relative to that in FG mice from week 5 onward, while body weight in the L-AFG group was increased relative to that in both FG and S-AFG mice between weeks 5 and 10 (Figure 1A). In line with these results, BFP was reduced in the S-AFG and increased in the L-AFG versus the FG group (Figure 1B), indicating differential effects of antibiotic treatment duration on fat accumulation.

These phenotypic differences were consistent with the metabolic parameters. Compared with the FG group, S-AFG mice displayed lower fasting blood glucose (FBG) and TG levels (Figures 1C,E), but L-AFG mice showed elevated FBG levels and reduced HDL-C levels (Figure 1F). These alterations suggest that short-term antibiotic treatment partially alleviates, while long-term treatment exacerbates, HFD-induced metabolic dysfunction.

As expected, the AG group (standard chow + short-term antibiotics) did not exhibit significant differences in body weight, fat percentage, or metabolic parameters compared with the BG group (standard chow + saline) (Figures 1A–F), indicating that short-term microbiota disruption alone has minimal metabolic impact under normal chow conditions.

In summary, these observations show that gut microbiota perturbation is differentially associated with HFD-induced obesity: short-term depletion exerts a protective effect, while long-term depletion exacerbates the obesity phenotype, which is accompanied by corresponding changes in metabolic indicators.

### 3.2 Divergent effects of short- and long-term microbiota remodeling on energy expenditure

To determine whether the divergent obesity phenotypes were associated with altered energy balance, both energy intake and expenditure were assessed. No significant differences in caloric intake were observed among HFD-fed groups (FG, S-AFG, L-AFG) (Figure 1G). We next evaluated energy expenditure. Whole-body metabolic rate was elevated in all HFD-fed groups compared with chow-fed controls and significant differences emerged after normalization to body weight (Figure 1H). Specifically, the S-AFG exhibited a higher weight-adjusted metabolic rate than the FG. However, the L-AFG group showed a lower metabolic rate than the S-AFG group, and did not differ from that of control groups. ANCOVA with body weight as a covariate revealed no significant effect of body weight on CL316243-stimulated energy expenditure (*p* = 0.978). However, after adjusting for body weight, a significant main effect of group was detected (*p* = 0.007, Partial Eta Squared = 0.317). Rectal temperature measured after CL316243 injection, a surrogate indicator of adaptive thermogenesis under β3-adrenergic stimulation, showed no significant difference between the S-AFG and FG groups. The L-AFG group exhibited lower rectal temperature (Figure 1I). Together, these results suggest that short-term antibiotic treatment enhances energy expenditure (primarily evidenced by elevated weight-adjusted CL316243-stimulated metabolic rate), whereas long-term treatment suppresses these processes under HFD conditions.

### 3.3 Short-term microbiota perturbation promotes, whereas prolonged depletion impairs, adipose tissue thermogenesis

**Figure 2:**
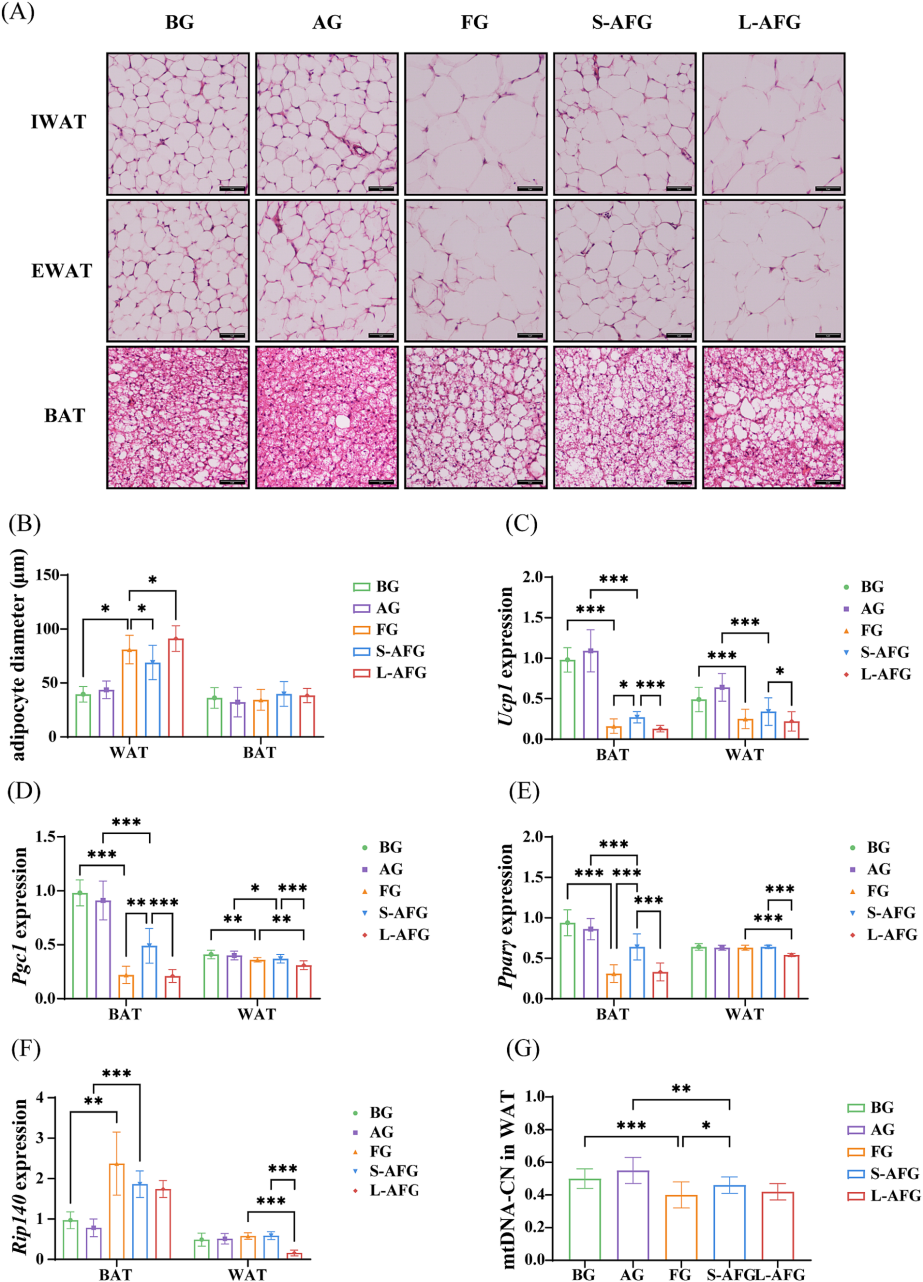
Short-term microbiota perturbation promotes, whereas prolonged depletion impairs, adipose tissue thermogenesis. **(A)** Representative hematoxylin and eosin (H&E) staining of inguinal white adipose tissue (IWAT), epididymal white adipose tissue (EWAT), and brown adipose tissue (BAT) sections. Scale bar = 50 μm. **(B)** Quantification of adipocyte diameter in WAT and BAT. **(C–F)** Relative mRNA expression of thermogenic genes (*Ppargc1a*, *Pparg*, *Ucp1*, *Nrip1*). **(G)** Mitochondrial DNA (mtDNA) copy number in WAT, normalized to β-actin. For panels **(A,B)** (histology), *n* = 20 mice per group. For panel **(C)** (mtDNA copy number) and panels **(D–G)** (qPCR), *n* = 20 mice per group (all 20 mice per group were used). *p < 0.05, **p < 0.01, and ***p < 0.001 versus the indicated groups.

Given the observed differences in energy expenditure, we evaluated thermogenic capacity in BAT and browning in WAT. Histological analysis further revealed marked differences in adipocyte morphology in WAT (Figure 2A). Compared with the FG group, adipocyte diameter was reduced in the S-AFG group, and it was further increased in the L-AFG group (Figure 2B). Adipocytes in the L-AFG group were larger than those in both FG and S-AFG groups, indicating enhanced lipid storage under prolonged microbiota disruption. Moreover, both S-AFG and L-AFG groups exhibited larger adipocytes than the antibiotic-only group (AG), suggesting that HFD remained the dominant driver of adipocyte hypertrophy. However, no significant differences in adipocyte diameter were observed among BAT groups.

Gene expression analysis further supported these findings. In BAT, thermogenic genes including *Ppargc1a*, *Pparg*, and *Ucp1* were upregulated in S-AFG mice compared with FG mice (Figures 2C–E). Conversely, these genes were significantly downregulated in L-AFG mice relative to S-AFG mice. In addition, the expression of *Nrip1*, a negative regulator of thermogenesis, was reduced in S-AFG mice compared with FG mice (Figure 2F). A similar pattern was observed in WAT. S-AFG mice showed increased expression of *Ppargc1a* and *Ucp1*, along with higher mitochondrial DNA copy number (mtDNA-CN), compared with FG mice (Figure 2G), indicating enhanced browning and mitochondrial biogenesis. In contrast, L-AFG mice exhibited reduced expression of *Nrip1*, *Ppargc1a*, *Pparg*, and *Ucp1* compared with both FG and S-AFG groups, suggesting markedly impaired browning capacity. Collectively, these data indicate that short-term antibiotic-induced microbiota remodeling promotes adipose tissue thermogenesis, whereas prolonged disruption suppresses this process.

### 3.4 Distinct trajectories of gut microbiota recovery underlie the opposing metabolic phenotypes

**Figure 3:**
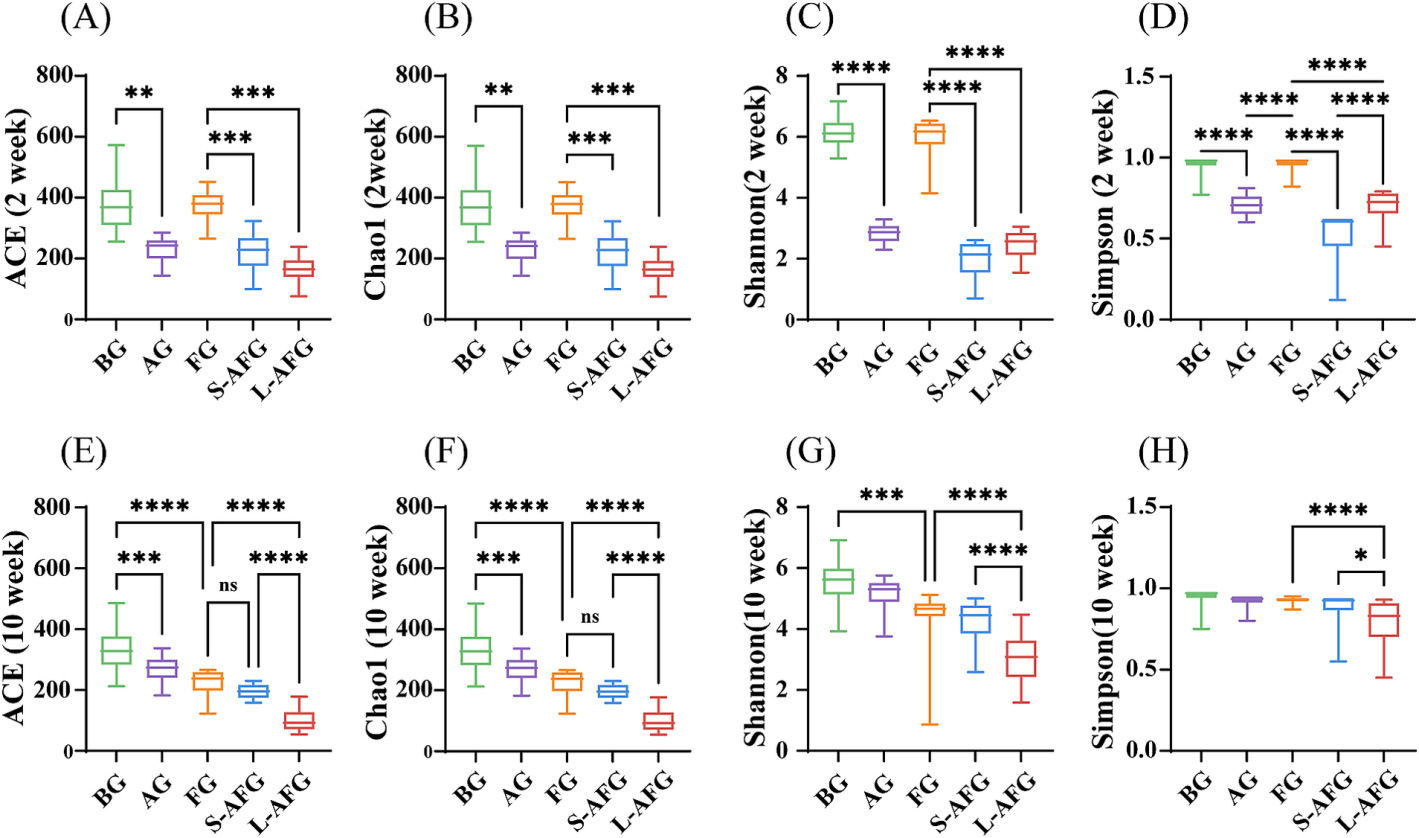
Distinct trajectories of gut microbiota recovery and compositional shifts underlie the opposing metabolic phenotypes. **(A–D)** Alpha diversity indices (ACE, Chao1, Shannon, Simpson) of fecal microbiota at the end of the 2-week antibiotic gavage period. **(E–H)** Alpha diversity indices at the termination of the experiment (week 10). For all panels, *n* = 10 mice per group (randomly selected from the 20 per group). Numerical data are provided in Supplementary Tables 5, 6. Data are expressed as the mean ± SD. *p < 0.05, **p < 0.01, and ***p < 0.001 versus the indicated groups.

**Figure 4:**
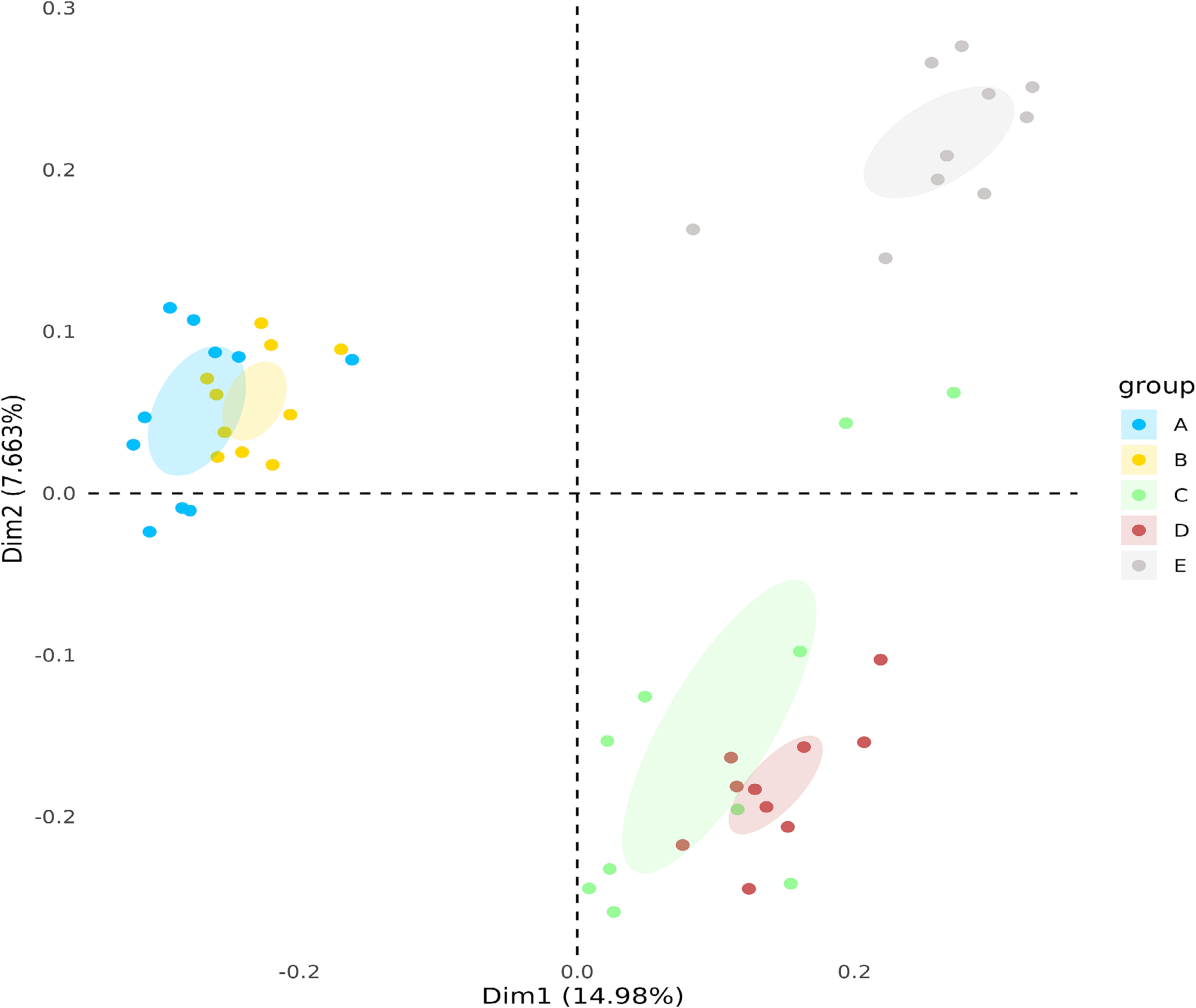
Principal coordinate analysis (PCoA) plot based on Bray-Curtis distances showing the overall microbial community structure at week 10. PERMANOVA confirmed a significant overall difference among groups (*F* = 3.939, *R*^2^ = 0.2593, *p* = 0.001). Pairwise comparisons: L-AFG (group E) differed from BG (group A), AG (group B), and FG (group C) (FDR-adjusted *p* < 0.01); S-AFG (group D) did not differ from FG (*p* = 0.316). *n* = 10 mice per group.

To investigate how different antibiotic regimens affected the gut ecosystem, fecal microbiota were analyzed at two time points. After the initial 2-week antibiotic treatment, alpha diversity (Abundance-based Coverage Estimator, ACE; Chao1, and Shannon indices) was markedly reduced in all antibiotic-treated groups (AG, S-AFG, L-AFG) compared with saline-treated groups (BG, FG) (Figures 3A–D). After the full 10-week experimental period, microbiota profiles diverged substantially. Alpha diversity in the S-AFG group was comparable to that in the FG group, suggesting partial recovery. On the contrary, the L-AFG group exhibited the lowest alpha diversity, with significantly reduced ACE, Chao1, and Shannon indices compared with all other groups (Figures 3E–H). The complete statistical data (mean ± SD) for all alpha diversity indices at both time points are provided in Supplementary Table 5 (week 2) and Supplementary Table 6 (week 10). Principal coordinate analysis (PCoA) at the endpoint directly showed substantial overlap between FG and S-AFG groups, and the L-AFG group formed a distinct cluster, clearly separated from all others (Figure 4, Dim1 was 14.98%, and Dim2 was 7.663%). PERMANOVA performed on Bray-Curtis distance matrices confirmed a significant overall difference in gut microbiota composition among the five experimental groups (*F* = 3.939, R^2^ = 0.2593, *p* = 0.001). ANOSIM further supported this separation (R = 0.7959, *p* = 0.001). Pairwise PERMANOVA comparisons with FDR adjustment revealed that the L-AFG group was significantly different from all other groups (vs. BG, *p* = 0.007; vs. AG, *p* = 0.007; vs. FG, *p* = 0.008; vs. S-AFG, *p* = 0.137, the latter not significant after correction). In contrast, the S-AFG group did not differ significantly from the FG group (*p* = 0.316), indicating partial recovery of the gut microbiota composition after short-term antibiotic withdrawal. The chow-fed groups (BG and AG) did not differ from each other (*p* = 1.000) nor from FG or S-AFG. These results are consistent with the PCoA clustering pattern (Figure 4) and indicate that long-term, but not short-term, antibiotic treatment induces a persistent and distinct dysbiotic state. Other pairwise results can be seen in Supplementary Table 4.

**Figure 5:**
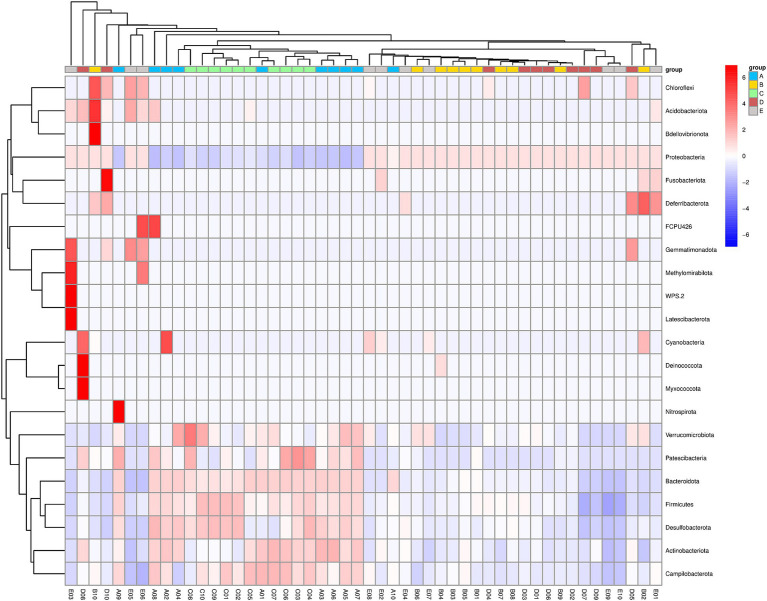
Heatmap of phylum-level relative abundance at week 2 (*n* = 10 per group). A, BG; B, AG; C, FG; D, S-AFG; E, L-AFG. Color scale: Z-scored abundance. Clustering shows separation between antibiotic-treated (B, D, E) and control groups (A, C).

**Figure 6:**
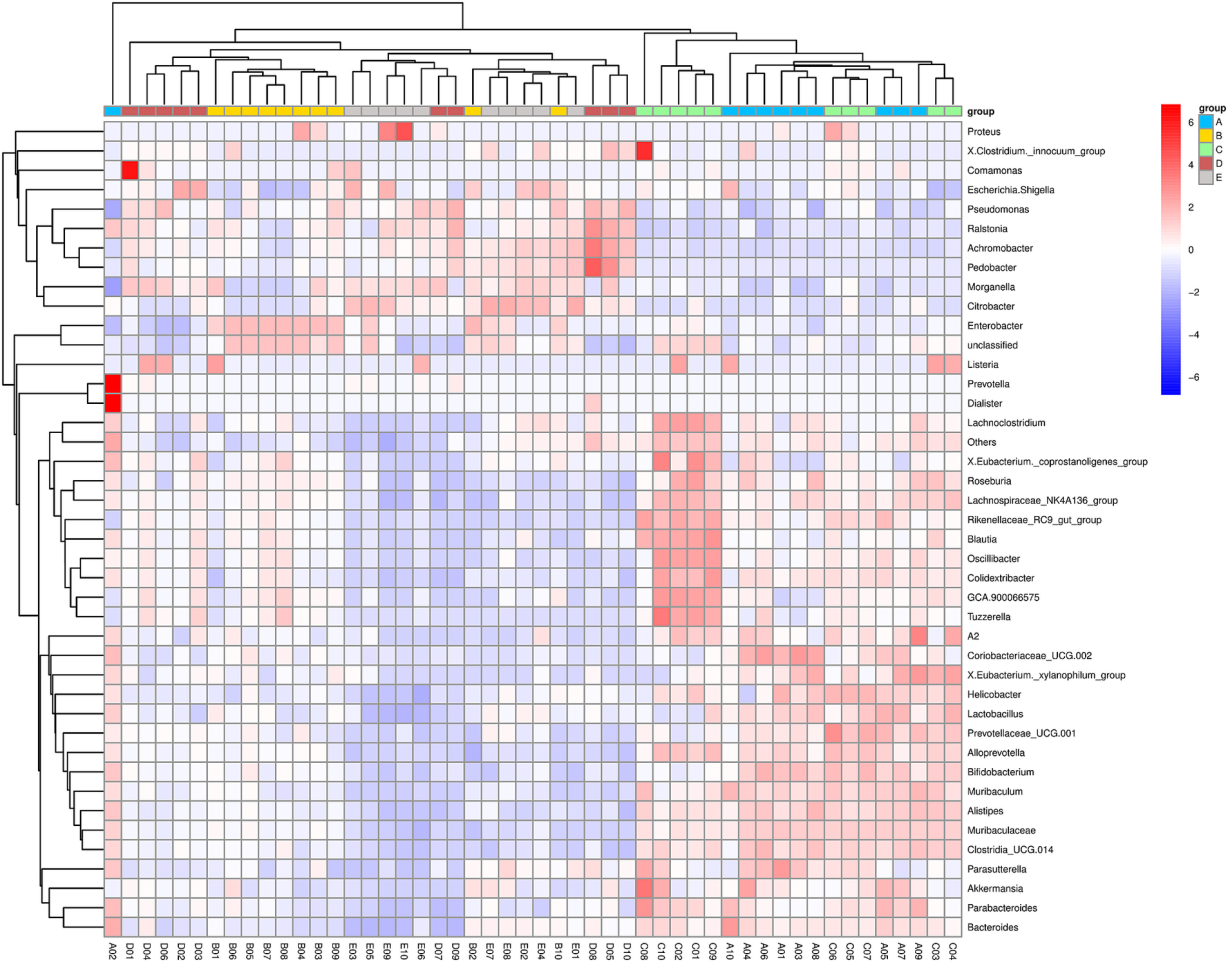
Heatmap of genus-level relative abundance at week 2 (*n* = 10 per group). A, BG; B, AG; C, FG; D, S-AFG; E, L-AFG. Color scale: Z-scored abundance. Clustering confirms distinct composition in antibiotic-treated groups.

**Figure 7:**
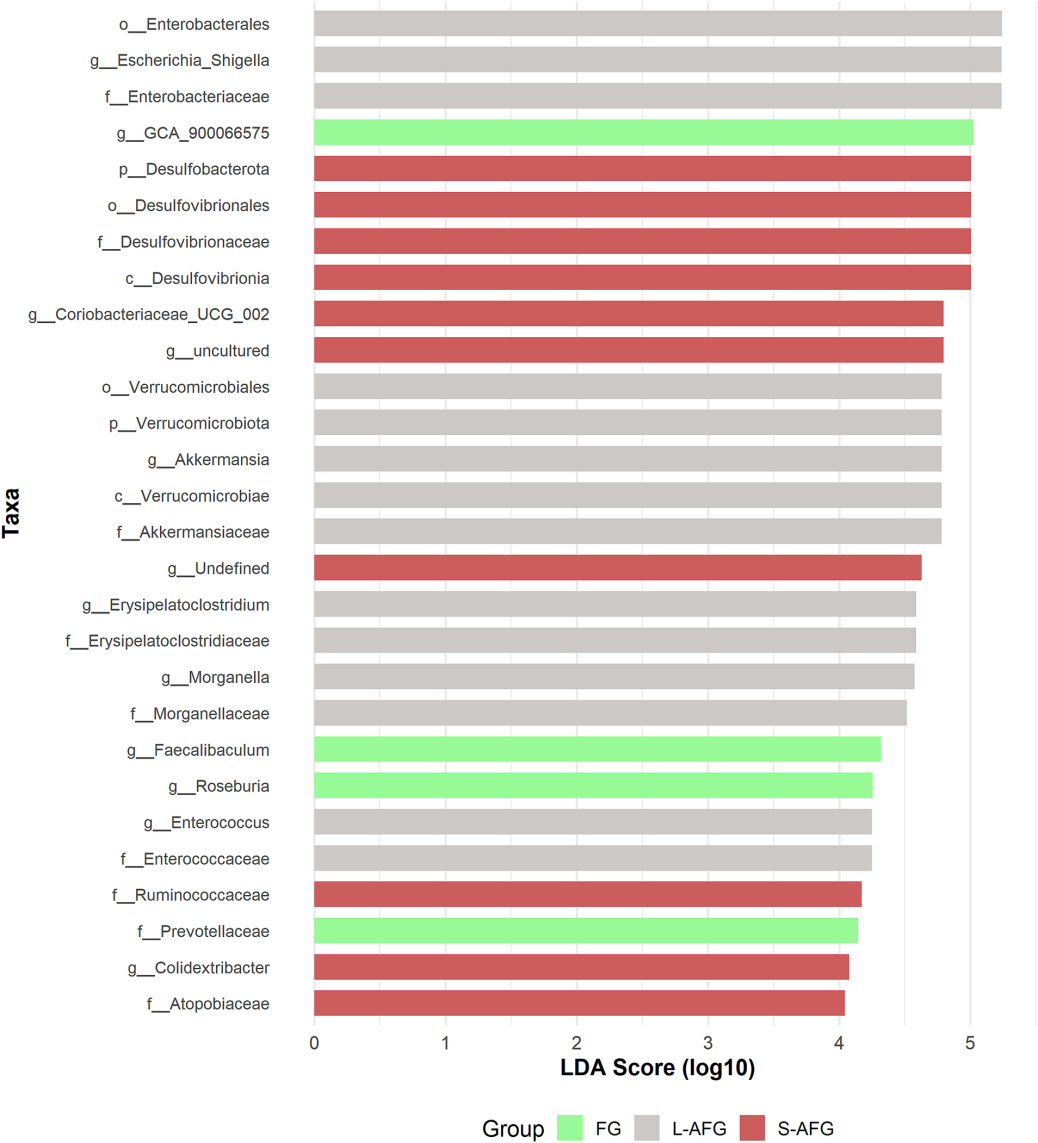
LEfSe bar plot (LDA > 2, *p* < 0.05) comparing FG, S-AFG, and L-AFG at week 10 (*n* = 10 per group). Bar length indicates LDA score.

At week 2, heatmap analysis revealed clear clustering separation between antibiotic-treated groups (AG, S-AFG, L-AFG) and control groups (BG, FG) at both the phylum (Figure 5) and genus (Figure 6) levels. To identify differentially abundant taxa, LEfSe analysis was applied to endpoint (week 10) fecal samples (LDA > 2.0, *p* < 0.05). In the S-AFG group, the most enriched taxon at the genus level was *Coriobacteriaceae_UCG-002* (LDA = 4.795, *p* = 3.92e-05). In the L-AFG group, *Escherichia-Shigella* (LDA = 5.239, *p* = 7.48e-06) was the most enriched at the genus level, with additional enrichments in Verrucomicrobiota (phylum, LDA = 4.779, *p* = 0.00039), Enterococcaceae (family, LDA = 4.250, *p* = 3.93e-05), and Erysipelatoclostridiaceae (family, LDA = 4.588, *p* = 2.10e-05) (Figure 7).

## 4 Discussion

In this study, we investigated the role of the gut microbiota in modulating energy metabolism and adipose tissue browning in HFD-induced mouse model of obesity. Our findings demonstrate that antibiotic-induced disruption of the gut microbiota is associated with a bidirectional effect on obesity development, depending on the duration of intervention. Specifically, short-term (2 weeks) microbiota depletion correlated with alleviated HFD-induced obesity, whereas long-term (10 weeks) depletion correlated with exacerbated metabolic dysfunction. These divergent effects were associated with differential regulation of energy expenditure, thermogenic gene expression in adipose tissues, and gut microbiota composition. Collectively, our data suggest that the gut microbiota influences host metabolic phenotype by modulating adipose tissue thermogenic capacity, thereby affecting systemic energy homeostasis.

### 4.1 Bidirectional effects of gut microbiota disruption on HFD-induced obesity

Obesity is fundamentally a disorder of energy imbalance, in which excess energy is stored as fat, particularly in WAT (Xiao et al., 2024). High-fat diets are well-established drivers of obesity in animal models (Hwalla and Jaafar, 2020). Consistent with previous studies, HFD feeding in our study induced significant increases in body weight, adiposity, and metabolic parameters. The gut microbiota has been recognized as a key regulator of host energy metabolism and nutrient absorption (Komodromou et al., 2024), yet the effects of antibiotic-induced microbiota disruption remain controversial (Gabriel and Ferguson, 2023; Sun et al., 2023). Our findings help reconcile these inconsistencies by demonstrating that the duration of microbiota perturbation is a crucial determinant of metabolic outcome. Short- and long-term microbiota perturbations exerted opposing metabolic effects, highlighting a temporal dependency in host–microbiota interactions. This bidirectional outcome aligns with prior studies reporting context-dependent metabolic consequences of antibiotic exposure, yet the temporal dimension has been largely overlooked (Mahana et al., 2016; Jayachandran and Qu, 2023). These discrepancies likely arise from differences in antibiotic composition, treatment duration, host developmental stage, and dietary context. Importantly, our results extend these observations by identifying treatment duration as a critical determinant that reconciles these seemingly contradictory outcomes. A possible explanation is that transient disruption allows for adaptive restructuring of the microbiota, whereas sustained perturbation leads to persistent dysbiosis and impaired host metabolic regulation. Interestingly, short-term antibiotic treatment had no discernible metabolic effect in chow-fed mice (AG vs. BG), suggesting that the protective effect observed in S-AFG mice requires an HFD background to manifest. This observation highlights the importance of dietary context in shaping the metabolic consequences of microbiota disruption.

Basal metabolic rate accounts for a major proportion of total energy expenditure, and adaptive thermogenesis is a key mechanism through which excess energy is dissipated (Theodorakis and Nikolaou, 2025). Under β3-adrenergic agonist stimulation, the measured metabolic rate reflects the maximal thermogenic capacity of the animals. After adjusting for body weight using ANCOVA, the S-AFG group still exhibited higher energy expenditure than the FG group, supporting that short-term microbiota disruption enhances energy expenditure independent of body weight differences. Our results under this standardized stimulation condition suggest that microbiota disruption modulates drug-stimulated energy expenditure, likely through regulation of thermogenic efficiency rather than energy intake. We acknowledge that the present study did not measure true resting basal metabolic rate. Our metabolic assessments were performed after CL316243 stimulation, which reflects drug-induced maximal thermogenic capacity rather than unstimulated basal energy expenditure. Future studies should evaluate basal thermogenesis under conditions without pharmacological stimulation to determine whether antibiotic-induced gut microbiota disruption affects basal metabolic rate.

Although rectal temperature is an indirect surrogate of thermogenesis (Abreu-Vieira et al., 2015), its directional consistency with CL316243-stimulated metabolic rate supports microbiota-dependent regulation of heat production. These findings are consistent with previous studies demonstrating that gut microbiota is required for proper thermogenic responses, particularly under conditions such as cold exposure (Zhou et al., 2024; Kazen et al., 2025). However, the exact contribution of rectal temperature as a functional readout can vary depending on measurement standardization. Future studies employing dynamic monitoring of rectal temperature during acute or chronic cold exposure, or non-invasive infrared thermography to assess BAT/WAT surface temperature (Chevalier et al., 2015), could provide more robust validation of these thermogenic differences and further elucidate the duration-dependent effects of microbiota disruption on adaptive thermogenesis.

### 4.2 Microbiota-driven regulation of adipose tissue thermogenic function

To further elucidate the mechanisms underlying altered energy expenditure, we examined thermogenic function in BAT and browning of WAT. The activation of BAT and induction of WAT browning increase energy expenditure through UCP1-mediated uncoupled respiration (Cypess and Kahn, 2010), processes regulated by transcriptional factors such as PPAR-gamma and PGC-1alpha (Takeda et al., 2023), while RIP140 acts as a negative regulator (Sharma et al., 2014). At the molecular level, microbiota perturbation is associated with changes in adipose tissue transcriptional networks governing mitochondrial function and uncoupled respiration. Prolonged dysbiosis may instead impair these regulatory circuits, potentially through chronic inflammatory signaling or loss of key microbial metabolites. These observations align with previous studies showing that microbiota depletion can impair thermogenic responses and that restoration of microbial balance can enhance energy expenditure (Guo et al., 2026). Collectively, these results suggest that the gut microbiota exerts a dynamic regulatory effect on adipose tissue function, with duration-dependent consequences for thermogenic capacity.

Beyond peripheral metabolic signaling, the gut microbiota may also regulate thermogenesis via the gut–brain–adipose axis. This neural regulatory pathway may contribute to the differential effects observed under short- versus long-term microbiota perturbation. In the present study, we employed a *β*3-adrenergic receptor agonist (CL316243) to induce adipose browning, thereby enabling the assessment of maximal thermogenic capacity under stimulated conditions. Given that the β3-AR agonist acts downstream of sympathetic outflow, it is possible that the microbiota may also influence central regulation of energy expenditure via gut-brain signaling pathways. Accumulating evidence indicates that microbiota-derived metabolites can modulate sympathetic nervous system activity (Barrea et al., 2019), which in turn drives β-adrenergic activation in brown and beige adipocytes (Bartness et al., 2010; Yan et al., 2025). Disruption of microbiota composition has been shown to alter hypothalamic inflammation and neural circuits controlling energy balance, thereby indirectly modulating adipose thermogenic responses (Picheswara Rao, 2026). However, our results on thermogenesis and under more physiological stimuli, such as cold exposure, warrant further investigation. It remains unclear whether the observed differences in thermogenic gene expression reflect an altered capacity to respond to sympathetic stimulation or a fundamental change in baseline thermogenic tone. While this study focused on the gut-adipose axis, the role of the central nervous system and the gut-brain axis in mediating these effects should be explored in future studies.

### 4.3 Dynamic alterations in gut microbiota diversity underlie the metabolic phenotypes

The distinct metabolic phenotypes observed in this study are closely linked to the temporal dynamics of gut microbiota recovery following antibiotic perturbation. Rather than a simple depletion–recovery process, previous evidence suggests that the gut microbiome may shift to an alternative stable state upon antibiotic perturbation, whereby the trajectory of recovery depends on the duration of disturbance (Dethlefsen and Relman, 2011). In the context of short-term antibiotic exposure, the microbiota may retain sufficient ecological redundancy to enable partial restoration of community structure and function. This resilience allows for the re-establishment of metabolically beneficial taxa and the recovery of key microbial functions, including the production of short-chain fatty acids and bile acid derivatives that support host energy expenditure and thermogenesis (Lee et al., 2025; Jäger et al., 2026). Although we did not directly measure short-chain fatty acids (SCFAs) or bile acids in this study, the increased abundance of *Coriobacteriaceae_UCG-002* in the S-AFG group (LDA = 4.795, *p* < 0.001, LEfSe) suggests a microbial shift that has been associated with favorable metabolic outcomes. Previous studies have shown that higher abundance of this genus is negatively correlated with serum and hepatic lipids and positively associated with the short-chain fatty acid hexanoic acid (Xu et al., 2023). Moreover, members of the Coriobacteriaceae family are known to participate in bile acid metabolism (Clavel et al., 2014). Collectively, these findings support that the enrichment of *Coriobacteriaceae_UCG-002*, alongside the restructuring of the gut microbiota after transient perturbation, represents a microbial signature linked to enhanced thermogenesis and attenuated obesity. However, we acknowledge that these associations do not prove causality, and future functional studies are needed to confirm its direct metabolic effects.

However, prolonged antibiotic intervention may exceed the resilience threshold of the microbial ecosystem, and a transition toward a stable but functionally compromised dysbiotic state can be observed. Such a state is characterized not only by reduced *α*-diversity but also by the loss of keystone taxa and functional capacity, which may not be readily reversible even after cessation of antibiotic exposure (Garza et al., 2025). In agreement with this, our study demonstrated that prolonged antibiotic treatment did induce such a persistent dysbiosis, which was associated with impaired host metabolic regulation, including reduced thermogenic capacity and mitochondrial dysfunction. Such long-lasting alterations may disrupt host–microbiota signaling networks, thereby contributing to sustained suppression of adipose tissue thermogenesis observed under chronic microbiota perturbation. Collectively, our findings support a model in which the duration of microbiota disruption plays a critical role in shaping whether the system undergoes adaptive remodeling or transitions into a maladaptive dysbiotic state, ultimately shaping host metabolic outcomes.

### 4.4 Limitations and future perspectives

Our study has several limitations. First, while we have identified a clear association between microbiota composition, adipose tissue function, and metabolic outcomes, the specific bacterial taxa and their functional metabolites responsible for these effects remain to be identified. Our 16S rRNA sequencing provided taxonomic profiles at the phylum and genus levels but lacked functional metagenomic data, preventing identification of specific microbial metabolic pathways (e.g., short-chain fatty acid or bile acid metabolism) that may mediate the observed effects on thermogenesis. Future studies should integrate metagenomics and metabolomics to pinpoint the key microbial species and their bioactive products that mediate the crosstalk with adipose tissue.

Second, our assessment of adipose tissue browning was limited to mRNA gene expression and mtDNA copy number at a single endpoint. While we observed significant changes in *Ppargc1a*, *Pparg*, and *Ucp1* expression, a more comprehensive analysis would require additional approaches. For example, protein-level quantification of UCP1 by Western blot or immunohistochemistry could confirm translational regulation. Dynamic imaging of BAT activity using infrared thermography or PET/CT would allow functional assessment of thermogenic capacity. Furthermore, exploring UCP1-independent thermogenic pathways, such as creatine cycling (Kazak et al., 2015), could reveal alternative mechanisms. Such approaches would provide deeper mechanistic insights.

Third, only male mice were included in our experiments. Recent studies have highlighted sex-specific effects on metabolic responses post-antibiotics. Schell et al. found that, following antibiotic exposure, female mice did not exhibit the reduction in energy expenditure or the increase in visceral adiposity observed in males (Schell et al., 2025). This suggests that sex is a non-negligible factor in future studies addressing the relationship between gut microbiota and energy expenditure.

Fourth, and most importantly, our study design does not allow us to infer causality. Although we observed strong associations between gut microbiota composition and thermogenic markers, we cannot conclude that the altered microbiota directly drives the thermogenic phenotypes. To establish causality, future studies should perform fecal microbiota transplantation (FMT) from donor mice in the S-AFG or L-AFG groups into germ-free or antibiotic-pretreated recipient mice, followed by assessment of thermogenic capacity.

Another limitation is the absence of a long-term antibiotic group on standard chow. In our pilot experiments, mice receiving the same cocktail under standard chow showed a survival rate below 60% by week 5, with severe weight loss, dehydration, and gastrointestinal intolerance. By contrast, HFD-fed mice tolerated the same regimen well (L-AFG group), likely due to the caloric surplus provided by the high-fat diet. To avoid excessive animal suffering, we did not include this group in the formal experiment, in accordance with the 3Rs principle. However, we acknowledge that the absence of this control limits our ability to conclude whether the detrimental metabolic effects of long-term antibiotic exposure are HFD-dependent. Future studies should explore optimized protocols (e.g., dose titration, intermittent dosing, or nutritional support) to enable such a comparison.

Finally, translating these findings to humans will require careful consideration. Antibiotic regimens in clinical practice are highly variable in terms of class, dose, and duration, and the long-term consequences of antibiotic-induced dysbiosis on energy metabolism remain a critical area for clinical investigation. The bidirectional effects observed in our study suggest that the timing and duration of antibiotic exposure may be important factors influencing metabolic outcomes in patients.

## 5 Conclusion

This study demonstrates that the gut microbiota is a crucial regulator of energy metabolism and adipose tissue function in HFD-induced obesity. The duration of microbiota disruption determines the metabolic outcome: transient perturbation enhances thermogenic capacity and energy expenditure, whereas chronic disruption leads to sustained dysbiosis, impaired thermogenesis, and exacerbated obesity. Our findings support a model in which the gut microbiota–adipose tissue axis plays a central role in metabolic regulation and represents a potential therapeutic target for obesity, particularly through duration-controlled microbiota modulation.

## Data Availability

The 16S rRNA sequencing raw reads for this study can be found in the NCBI Sequence Read Archive (SRA) under BioProject ID PRJNA1449050. Additional raw data supporting the conclusions of this article will be made available by the authors, without undue reservation.
