# Supplementary material for: Duration-dependent gut microbiota disruption drives opposing effects on adipose thermogenesis in obese mice
Source: Front Microbiol. 2026 Jul 22;17:1860759. doi: 10.3389/fmicb.2026.1860759 (PMC13437555; doi:10.3389/fmicb.2026.1860759)
Supplement: Supplementary file 1 [file Table_1.DOCX]

Supplementary Material

# 1 Supplementary Figures and Tables

## 1.1 Supplementary Tables

The relevant TSE PhenoMaster instrumentation details (scanner, analytical sensors, flow control) are supported by Supplementary Table 1.

Supplementary Table 1

| Parameter | Specification |
| --- | --- |
| Instrument model | PhenoMaster (TSE Systems, Germany) |
| Measurement type | Open-flow indirect calorimetry |
| Number of chambers | 4 |
| Chamber volume | 4.9 L |
| Ambient temperature | 30 ± 1°C |
| Light/dark cycle | 12 h/12 h |
| Flow rate per chamber | 0.62 L/min |
| O₂ sensor type | Zirconia oxide / electrochemical |
| O₂ sensor resolution | 0.01% |
| CO₂ sensor type | Infrared absorption |
| CO₂ sensor resolution | 0.01% |
| Sampling interval | 27 min per cage |
| RER calculation | VCO₂/VO₂, Average RER values for all mice ranged from 0.70 to 0.86 |
| Activity monitoring | InfraMot, 3D infrared beam frames |
| Spatial resolution | 7 mm |

Primers used for complementation can be seen in Supplementary Table 2. All primers used in this experiment were synthesized by Tsingke Biotechnology Co., Ltd. (Beijing, China).

Supplementary Table 2

| Name of primers | Primer sequence |
| --- | --- |
| Mouse Pparg-1F | CGAGAAGGAGAAGCTGTTG |
| Mouse Pparg-1R | TCAGCGGGAAGGACTTTA |
| Mouse Ucp1-1F | CCGGCTTAATGACTGGAG |
| Mouse Ucp1-1R | GTAGCGGGGTTTGATCC |
| Mouse Ppargc1a-1F | TTCCAACAAACACATGCAC |
| Mouse Ppargc1a-1R | CGCATTTCTAAAGCACCAG |
| Mouse Nrip1-1F | AACGCAGAACGCACATC |
| Mouse Nrip1-1R | CAGACACCCCTTTGGAGA |
| Mouse M-GAPDH-F | CAGTGGCAAAGTGGAGATTGTTG |
| Mouse M-GAPDH-R | TCGCTCCTGGAAGATGGTGAT |
| Mouse M-Actb-1F | CCACCATGTACCCAGGCATT |
| Mouse M-Actb-1R | CAGCTCAGTAACAGTCCGCC |
| Mouse MT-ND1-F | TGGGTACAATGAGGAGTAGG |
| Mouse MT-ND1-R | GGAGTAATCCAGGTCGGT |

Supplementary Table 3. Detailed parameters of 16S rRNA gene sequencing and bioinformatics analysis

| Parameter | Specification |
| --- | --- |
| Sample source | Fresh mouse feces |
| DNA extraction kit | TIANGEN Fecal Genomic DNA Extraction Kit (DP328-02, TIANGEN, Beijing, China) |
| Amplified region | V3‑V4 hypervariable region of bacterial 16S rRNA gene |
| Forward primer | 341F (5′‑CCTACGGGNGGCWGCAG‑3′) |
| Reverse primer | 805R (5′‑GACTACHVGGGTATCTAATCC‑3′) |
| Sequencing platform | Illumina NovaSeq 6000 (paired‑end, 2 × 250 bp or 2 × 300 bp) |
| Raw reads per sample | 70,000 – 90,000 (before filtering) |
| Quality filtering tool | Fastp v0.20.0 (low‑quality threshold 40, ambiguous bases >5 removed) |
| Read merging tool | FLASH v1.2.7 (minimum overlap 10 bp, max mismatch ratio 0.1) |
| Denoising and ASV inference | DADA2 algorithm within QIIME 2 (2023.2) |
| Chimera removal | Consensus method in DADA2 |
| ASV count | 2,347 ASVs across all samples |
| Taxonomic reference database | SILVA 138 (http://www.arb-silva.de) |
| Classifier | Naïve Bayesian (QIIME 2, confidence threshold 0.8) |
| Rarefaction depth | 31,000 reads (based on plateau of rarefaction curves and minimum library size) |
| Software for alpha diversity | R v4.2.1 (vegan v2.6‑4) |
| Software for beta diversity | R v4.2.1 (phyloseq v1.42.0) |
| Normalization for beta diversity | DESeq2 variance‑stabilizing transformation |
| PERMANOVA permutations | 999 |

Supplementary Table 4. PERMANOVA results for gut microbiota beta diversity (Bray‑Curtis distance)

| Comparison | Df | SumOfSqs | R² | F | P‑value (raw) | FDR‑adjusted P |
| --- | --- | --- | --- | --- | --- | --- |
| Overall (5 groups) | 4 | 4.231 | 0.2593 | 3.939 | 0.001 | – |
| Residuals | 45 | 12.085 | 0.7407 | – | – | – |
| Total | 49 | 16.316 | 1.000 | – | – | – |
| Pairwise: BG vs. AG | – | – | – | – | 0.7335 | 1.000 |
| BG vs. FG | – | – | – | – | 0.7349 | 1.000 |
| BG vs. S‑AFG | – | – | – | – | 0.0633 | 0.316 |
| BG vs. L‑AFG | – | – | – | – | 0.00075 | 0.007 |
| AG vs. FG | – | – | – | – | 0.9758 | 1.000 |
| AG vs. S‑AFG | – | – | – | – | 0.0510 | 0.306 |
| AG vs. L‑AFG | – | – | – | – | 0.00071 | 0.007 |
| FG vs. S‑AFG | – | – | – | – | 0.0662 | 0.316 |
| FG vs. L‑AFG | – | – | – | – | 0.00098 | 0.008 |
| S‑AFG vs. L‑AFG | – | – | – | – | 0.0196 | 0.137 |

Note: Pairwise PERMANOVA was performed with 999 permutations and Benjamini‑Hochberg FDR correction. Bold indicates significance (adjusted *P* < 0.05).

Supplementary Table 5. Alpha diversity indices at the end of the 2‑week antibiotic gavage period (n = 10 per group, mean ± SD)

| Group | ACE | Chao1 | Shannon | Simpson |
| --- | --- | --- | --- | --- |
| BG | 371.08±114.12 | 370.52±114.27 | 5.81±1.02 | 0.95±0.06 |
| AG | 231.88±36.42 | 231.42±36.41 | 2.77±0.36 | 0.70±0.09 |
| FG | 376.64±66.79 | 376.55±66.66 | 5.95±0.72 | 0.95±0.05 |
| S-AFG | 220.20±76.03 | 219.64±75.92 | 1.97±0.55 | 0.49±0.15 |
| L-AFG | 160.77±59.25 | 160.61±59.33 | 2.48±0.50 | 0.69±0.09 |

Supplementary Table 6. Alpha diversity indices at the termination of the experiment (week 10) (n = 10 per group, mean ± SD)

| Group | ACE | Chao1 | Shannon | Simpson |
| --- | --- | --- | --- | --- |
| BG | 319.81±86.60 | 319.13±86.43 | 5.49±0.90 | 0.92±0.07 |
| AG | 257.46±48.86 | 257.12±48.67 | 5.02±0.60 | 0.91±0.05 |
| FG | 198.19±58.39 | 197.84±58.25 | 4.50±0.99 | 0.88±0.14 |
| S-AFG | 175.61±40.39 | 175.12±40.22 | 4.00±0.83 | 0.83±0.13 |
| L-AFG | 89.30±39.17 | 89.02±39.05 | 2.87±1.01 | 0.73±0.17 |
